# Danggui-Shaoyao-San Attenuates Cognitive Impairment *via* the Microbiota–Gut–Brain Axis With Regulation of Lipid Metabolism in Scopolamine-Induced Amnesia

**DOI:** 10.3389/fimmu.2022.796542

**Published:** 2022-05-19

**Authors:** Piaoxue Liu, Xun Zhou, Haoran Zhang, Rui Wang, Xiaolang Wu, Wenxuan Jian, Weirong Li, Dongsheng Yuan, Qi Wang, Wei Zhao

**Affiliations:** ^1^ Science and Technology Innovation Center, Guangzhou University of Chinese Medicine, Guangzhou, China; ^2^ Gastrointestinal Surgery Department, The First Affiliated Hospital of Jinan University, Guangzhou, China

**Keywords:** Danggui-Shaoyao-San, gut microbia, lipid metabolism, gut–brain axis, cognitive disorder

## Abstract

Danggui-Shaoyao-San (DSS) has a long history of being used as a traditional medicine (TCM) and has been reported to show therapeutic effects in alleviating the symptoms of cognitive impairment. The purpose of this study was to investigate whether DSS treatment attenuates cognitive impairment *via* the microbiota–gut–brain axis in scopolamine-induced amnesia. In this work, we first performed the Morris water maze (MWM) test and novel object recognition (NOR) test to evaluate the memory function of treated C57BL/6N mice. Then we evaluated 16S rRNA for gut microbiota analysis, as well as assessment of blood–brain barrier function and intestinal barrier function and lipid metabolism analysis on tissues from different groups. We hypothesised that DSS may affect brain function and behavior through the gut–brain axis in a bidirectional interplay with both top-down and bottom-up regulation. Furthermore, in order to confirm whether intestinal flora plays a crucial role in scopolamine-induced amnesia, C57BL/6N mice were treated with fecal microbial transplantation (FMT), and then behavioral tests were performed. The mice’s feces were simultaneously evaluated by 16S rRNA analysis. The result supported that the FMT-induced improvement in cognitive function highlights the role of the gut microbiota–brain axis to mediate cognitive function and behavior. Besides theses works, more findings indicated that DSS altered lipid metabolism by activating LXR-PPAR-γ and repaired mucosal barrier dysfunction assessed with a broad range of techniques, which attenuated cognitive impairment *via* the microbiota–gut–brain axis.

## Introduction

Neurodegenerative diseases related to cognitive disorder and dysmnesia are the main cause of the dramatic decline in morbidity, quality of life, and healthcare costs in an increasingly aging population (1). Meanwhile, a revolution has occurred in biomedicine with the realization that gut microbiota and microbiomes have a role in neurodevelopment, neuroinflammation, and behavior. Over the past 5 years, a growing body of research has focused on illuminating the bidirectional communication pathways between gut bacteria and the central nervous system (CNS), such as the microbiota (2)–gut–brain axis (3, 4), and dysregulation of this axis has been increasingly implicated in the pathophysiology of Alzheimer’s disease (5). Much recent evidence suggests that the gut microbiome plays an important role in the pathogenesis of neurological disorders (6–8). Studies have shown the implication of the gut–brain axis in an integrated network wherein the microbiome and the central nervous system cross-talk *via* endocrine, immune, and neural signaling pathways in various aspects of host health and diseases (9, 10). Recent studies have reported an altered gut microbiome in patients with mild cognitive impairment (MCI), dementia, and Alzheimer’s disease (5, 11–14) as well as in multiple AD animal models (15–17). This evidence suggests that gut microbiota communicates with the brain and vice versa through complex bidirectional communication systems—the gut–brain axis (18, 19).

For thousands of years, traditional Chinese medicine (TCM) has played an irreplaceable role in the Chinese medical system (20), and TCM has unique advantages in treating complex diseases such as dysmnesia. The holistic view of (TCM) regarding the treatment of multiple components and multiple targets provides a bright prospect for the prevention and treatment of cognitive disorders (21). TCM can exert its effect by operating in the gut microbiota and is a natural intestinal microecological regulator, and the gut microbiota has emerged as a novel and important field for understanding TCM (22, 23). Emerging evidence has indicated that the interactions between TCM and gut microbiota could lead to changes in structural or functional modulation of gut microbiota (24, 25).

A well-known TCM formulation, Danggui-Shaoyao-San (DSS), also called Dangguijakyak-san or Toki-shakuyaku-san in Japanese, has been widely used in the treatment of various neurodegenerative diseases in China for more than 2,000 years (26). Analysis by HPLC-DAD-ESI-MS/MS revealed that DSS contains gallic acid, ferulic acid, albiflorin, Z-ligustilide, senkyunolide I, monoterpene glycosides, phenolic acids, phthalides, sesquiterpenoids, triterpenes, paeoniflorin, benzoic acid, coniferyl ferulate, senkyunolide A, Z-butylidenphthalide, 3-butylphthalide, atractylcnolide II, atractylcnolide I, levistolide A, and so on (27). Clinically, a large amount of evidence supports the therapeutic effect of DSS on cognitive impairment through limiting neuronal damage, possessing antioxidant capability, enhancing cognitive behavior, reducing cell apoptosis in the hippocampus, and inhibiting neuroinflammation (26, 28–30). However, further investigation of the molecular mechanism of the therapeutic effect of DSS on cognitive impairment is required in order to propose effective treatment strategies.

In a recent investigation, DSS was shown to markedly regulate gut microbiota and lipid metabolism (31). Compelling evidence supports that lipid metabolism and the pathogenesis of cognitive disorder are closely linked. In observational studies, high levels of serum cholesterol have been associated with an increased risk of Alzheimer’s disease (32). Lipids are essential in maintaining brain function. It is reported that lipid profiles can be altered in dementia brains as compared to normal brains. Nutriment has the potential to ameliorate cognitive decline which affects lipid metabolism levels as a whole. The gut microbiota also serve as a source of beneficial lipids; we should not ignore to regulate the disturbance flora (33).

Taken together, we hypothesized that the effect of DSS on ameliorating cognition deficits and pathology may be *via* the microbiota–gut–brain axis with the regulation of lipid metabolism and inflammation in dementia mice. In the present study, we focus on understanding changes in the microbiota–gut–brain axis and the lipid metabolism after DSS administration.

## Materials and Methods

### Materials and Reagents

The 6 raw herbs of DSS were purchased from Kangmei Pharmaceutical Limited Company (Guangzhou, China). Donepezil hydrochloride (D849374) and scopolamine hydrobromide trihydrate (S860151) were from Macklin (Shanghai, China); FITC-dextran (^#^46944) was from Sigma system (ChemiDoc MP, Bio-Rad, California, USA); total cholesterol Assay Kit (A111-1-1) and Triglyceride Assay Kit (A110-1-1) were from Nanjing Jiancheng; and Malondialdehyde (MDA) ELISA Kit (JL13339), Adiponectin (ADPN) Kit (JL20696D), Low-Density Lipoprotein Cholesterol (LDL-C) Kit (JL20313), High-Density Lipoprotein Cholesterol (HDL-C) Kit (JL20356), Tumour Necrosis Factor-α Kit (TNF-α) (JL10484) and Interleukin 6 (IL-6) Kit (JL20313) were from Jiang Lai Biological (Shanghai, China). Antibodies included rabbit anti-ZO-1 (PB9234, Boster, Pleasanton, CA, USA), rabbit anti-OCLN (A01246-2, Boster), rabbit anti-OCLN (#91131, CST, Danvers, MA, USA) in brain immunofluorescence, rabbit anti-ZO-1 (#13663, CST) in brain immunofluorescence, rabbit anti-PPAR-γ (^#^2435, CST), Cy3-labelled Goat Anti-Rabbit IgG (H+L) (A0516, Beyotime, Shanghai, China), rabbit anti-LXR alpha+beta (ab21669, Abcam, Cambridge, MA, USA), rabbit anti-actin (^#^2118, CST), and rabbit anti-GAPDH (AP0063, Bioworld, St. Louis Park, MN, USA).

### DSS Preparation

*Angelica sinensis* (Oliv.) Diels, *Paeonia lactiflora* Pall., *Ligusticum chuanxiong* Hort., *Poria cocos* (Schw.) Wolf., *Alisma plantago-aquatica* Linn., and *Atractylodes macrocephala* Koidz. were mixed with a dose proportion of 3:16:8:4:8:4. Mixed in this ratio, the 6 herbs were soaked in distilled water for 1 h, boiled for 0.5 h, and then simmered for 1 h. After the filtrate was collected, distilled water was added to extract for 1 h. The final filtrate was blended and concentrated to 1 g/ml, which was eventually freeze-dried with a lyophiliser and sealed at -20°C.

### Animals and Treatments

Five-week-old male C57BL/6N mice raised under SPF conditions were purchased from the Guangdong Medical Experimental Animal Center (Guangzhou, China). They were housed in a specific pathogen-free, temperature- and humidity-controlled environment (22 ± 2°C, 50 ± 5% humidity) with a standard 12-h light/dark cycle. These mice were given access to food and water and allowed to acclimatize to the animal facility environment for a week before being used for experimentation. Experimental protocols had been obtained approval from the Animal Experimentation Committee at Guangzhou University of Chinese Medicine and the experimental protocols were conducted in accordance with the National Research Council Guide for the Care.

### Groups and Drug Administration

After a week of adjustable feeding, the C57BL/6N mice were randomly divided into 4 groups (10 mice/group): (1) control group, CON (0.9% saline, 10 ml/kg/day), (2) scopolamine-induced group, SCO (0.9% saline, 3 mg/kg/day), (3) donepezil treatment group, DPZ (3 mg/kg/day), and (4) DSS treatment group, DSS (4.8 g/kg/day). At first, mice were orally treated with DSS pre-administration for 1 week. Next, cognitive impairment was induced by scopolamine given in saline solution for 2 weeks except for those in the CON group, while each group was individually administered according to the above grouping. After this treatment, behavioral experiments were conducted on the mice and those were then sacrificed in order to yield samples for subsequent experiments.

### Morris Water Maze Test

The pool was divided equally into 4 quadrants, with a platform hidden about 1 cm below the water. After administration for 2 weeks, all mice were allowed adaptive training for the Morris water maze (MWM) test: each of the mice was given 1 min to find the platform and allowed to stay there for 20 s before being removed from the platform. Each mouse was trained 3 times. In the next 5 days, each of the mice was trained to find the platform at its then-present location. On the last day, all of the mice were allowed to swim in the pool freely and to find the platform, which was removed on the 6th day. The swimming path and the time spent finding the platform for each mouse were detected by a camera, and the crossing times of the platform were measured by software.

### Novel Object Recognition

We performed a novel object recognition (NOR) test. On the first day, mice were conditioned to move freely in an empty plastic box for 5 min. After 24 h of training, the mice were placed back in the same box with 2 objects of the same size and shape and allowed to explore freely for 5 min. The objects were cleaned thoroughly between trials to avoid olfactory cues. After 24 h, one of the objects was replaced with a new object of the same size but different shape, and the mice were allowed to explore freely for another 5 min. Simultaneous video and tracking documented the detection time of each object. Detection was defined as the mouse facing the object, sniffing or touching with the nose, and with the recorded distance from the nose to the object less than or equal to 2 cm. The calculation method was defined as the percentage of the time a mouse explored the new object or location over the total time the mouse explored the 2 objects or locations.

### Blood and Tissue Sample Collection

At the end of the experiments, the mice were not fed overnight and then anaesthetized with sodium pentobarbital (50 mg/kg), and blood was obtained by cardiopuncture. These blood samples were centrifuged at 2,500 rpm for 10 min to collect the serum samples, which were immediately frozen at -80°C for biochemical assays. Hippocampus, jejunum, and colon tissues were dissected for histopathology, immunofluorescence staining and Western blot.

### 16s rRNA Gene Sequence Analyses

Fresh stool samples from mice were collected in disinfected tubes, immediately frozen in liquid nitrogen upon collection before the mice were sampled, and stored at -80°C until analysis. The PCR primer was designed against the conserved region to target the variable region of the 16S/ITS2 rDNA gene. After 35 cycles of PCR, sequencing adapters and barcodes were added for amplification. PCR amplification products were detected by 1.5% agarose gel electrophoresis. The target fragments were recovered using the AxyPrep PCR Cleanup Kit. The PCR product was further purified using the Quant-iT Pico Green dsDNA Assay Kit. The library was quantified on the Promega QuantiFluor fluorescence quantification system. The pooled library was loaded on an Illumina platform using a paired-end sequencing protocol (2 × 250 bp) by LC-Bio Sciences.

Paired-end reads were assigned to samples based on their unique barcode and truncated by cutting off the barcode and primer sequence. Paired-end reads were merged using FLASH (v1.2.8) (for 16S)/PEAR (v0.9.6) (for ITS2). Quality filtering on the raw reads was performed under specific filtering conditions to obtain the high-quality clean tags according to fqtrim (v0.94). Chimeric sequences were filtered using VSEARCH software (v2.3.4). After dereplication using DADA2, we obtained a feature table and feature sequence. Alpha diversity and beta diversity were calculated by QIIME2, by which the same number of sequences were extracted randomly by reducing the number of sequences to the minimum of some samples, and the relative abundance (X bacterial count/total count) was used in bacteria taxonomy. Alpha diversity and beta diversity were analyzed by the QIIME2 process, and pictures were drawn by R (v3.5.2). The sequence alignment of species annotation was performed by Blast, and the alignment databases used were SILVA and NT-16S.

### Histological Analysis of Intestine

Paraffin sections were used for haematoxylin and eosin (H&E) staining and immunofluorescence staining, which followed the manufacturer’s protocol. Full-thickness sections of jenjum and colon were excised, dissected longitudinally, fixed immediately in 4% paraformaldehyde solution, and embedded in paraffin. Samples were cut into 5-µm-thick sections, mounted on slides, and stained with haematoxylin and eosin (H&E). The epithelial morphological characteristics were observed microscopically (Nikon Eclipse 80i). In the same way, the jejunum was cut into 5-µm-thick sections, mounted on slides, and stained with immunofluorescence staining. After washing for 10 min with TBS, samples were blocked with TBS for 2 h containing 1% w/v bovine serum albumin (BSA) to prevent non-specific binding during immunohistochemical analysis of tight-junction protein expression and then incubated overnight at 4°C with anti-ZO-1, OCLN antibodies (Boster Biological Technology, China) at a dilution of 1:500. The next day, after washing 3 times with TBS for 15 min, the samples were treated with Cy3-labelled Goat Anti-Rabbit IgG (H+L) at a ratio of 1:500. The samples were incubated at room temperature for 1 h and washed 3 times with TBS. The processed slides were observed under a fluorescence microscope (Leica TCS SP8) and a Leica confocal microscope.

### Histological Analysis of the Hippocampus

The mice were anesthetized, transcardially perfused with 0.05 M phosphate-buffered saline (PBS), and fixed in cold 4% paraformaldehyde (PFA). Brain tissues were removed, postfixed in 0.05 M PBS containing 4% PFA overnight at 4°C, and cryopreserved in 30% sucrose in for cryoprotection. Next, the brain tissues were embedded in Tissue-Tek OCT and cut into coronal frozen sections (30 µm). Subsequently, we performed Nissl staining on the sections. Sections were microscopically examined using Nikon Eclipse 80i, and images were captured for analysis. Likewise, the brain tissues were cut into 30 µm coronal frozen sections, placed on the slides, and analysed by immunofluorescent staining.

### Intestinal Permeability

After the last drug administration, the mice were not fed overnight and gavaged FITC-dextran (40 mg/100 g phosphate-buffered saline) to measure the concentration of serum glucanhydride. Blood samples were collected from the eyeball, and the blood was diluted 5 times in PBS. Fluorescence values were read using a Nikon fluorescence microscope (excitatory wavelength: 480 nm, emission wavelength: 520 nm) and measured with a 96-well plate (excitation: 485 nm, emission: 528 nm) to calculate the concentration of FITC-dextran. The standard concentration range of dextran prepared with PBS was 0–250 g/ml FITC. Detection of the level of dextran in serum was conducted in order to observe the destruction of the intestinal barrier.

### Blood Serum Analysis

Blood was sampled from the retroorbital space after the animals were anaesthetised with sodium pentobarbital or direct cardiac puncture immediately after death. Plasma levels of high-density lipoprotein (HDL), low-density lipoprotein (LDL) and malondialdehyde (MDA) were measured by Enzyme-Linked Immunosorbent Assay (ELISA) using LDL-C ELISA Kit, HDL-C ELISA Kit and MDA ELISA Kit, (J&L Biological). For instance, a monoclonal antibody specific for mouse LDL-C was coated onto the microplates. Wells were incubated with test samples as well as HRP (horseradish peroxidase) for 1 h at 37°C of incubation and washed 5 times. Then, wells were incubated in 100 µl of substrate solution for 15 min and stopped with a stop solution (50 µl). Finally, the values of each well were measured at 450 nm.

### Lipid Parameters

The brain tissues were placed in the phosphate-buffered saline (PBS) solvent with the homogeniser machine at 3,000 rpm at 4°C for 15 min. The cleared lysates were obtained by centrifugation at 3,000 rpm at 4°C for 30 min. The levels of total cholesterol (TC) and triglyceride (TG) were measured using commercial kits (Total Cholesterol Assay Kit and Triglyceride Assay Kit, Nanjing Jiancheng). Finally, 10 µl of supernatant was added to a 96-well plate and the absorbance was measured at 510 nm.

### Fecal Microbial Transplantation Treatment

After a week of adjustable feeding, the C57BL/6N mice were randomly divided into 4 groups (10 mice/group): (1) control group, CON (0.9% saline, 10 ml/kg/day), (2) scopolamine-induced group, SCO (0.9% saline, 3 mg/kg/day), (3) donepezil treatment group, DPZ (3 mg/kg/day), and (4) fecal microbial transplantation (FMT) treatment group. At first, the FMT group were pretreatment with broad-spectrum antibiotics for 3 days. For the microbiota suspension preparation, several fresh feces pellets (80–100 mg) were collected from DSS administration mice using sterile tubes and resuspended with a vortex in 600 μl PBS. After resuspension, tubes containing the feces in PBS were centrifuged at 3,000 rpm for 3 min to remove insoluble material. Mice were given 100 μl of the microbiota suspension three times a week. Cognitive impairment was induced by scopolamine given in saline solution for 2 weeks except those in the CON group, while each group was individually administered according to the above grouping. After this treatment, animal behavioral studies were conducted until natural death or sacrifice.

### Western Blot Analysis

Colon and hippocampal tissues were sonicated with RIPA lysate (P0013C, Beyotime) containing 1× protease and phosphatase inhibitors. The cleared lysates were obtained by centrifugation at 3,000 rpm at 4°C for 15 min. The protein concentration was quantified using the Thermo Fisher Scientific BCA Protein Analysis Kit with bovine serum albumin as a standard and then mixed with the loading buffer, heated at 100°C for 10 min; equivalent amounts of protein from each sample were separated by 10% SDS-PAGE and transferred onto polyvinylidene fluoride membranes (Millipore, Darmstadt, Germany). Subsequently, the membranes were blocked in 5% BSA, probed overnight at 4°C with primary antibodies, and then incubated with HRP-conjugated secondary antibodies. The signals were detected with an enhanced chemiluminescence system (ChemiDoc MP, Bio-Rad, Hercules, California, USA). The immunoreactive bands were quantified *via* densitometry using ImageJ (Version 1.50b, National Institutes of Health, Bethesda, MD, USA) and standardized to actin and were expressed as fold changes relative to the control value.

### Statistics

All statistical analyzes were performed using GraphPad Prism software version 8, and all data are expressed as the mean ± standard deviation. For data with a normal distribution and homogeneity of variance. One-way ANOVA was used to evaluate significant differences between the 2 groups. *p <* 0.05 was considered significant; * *p <* 0.05, ** *p <* 0.01, *** *p <* 0.001.

## Results

### DSS Treatment Ameliorated Cognitive Impairment in Scopolamine-Induced Mice

**Figure 1:**
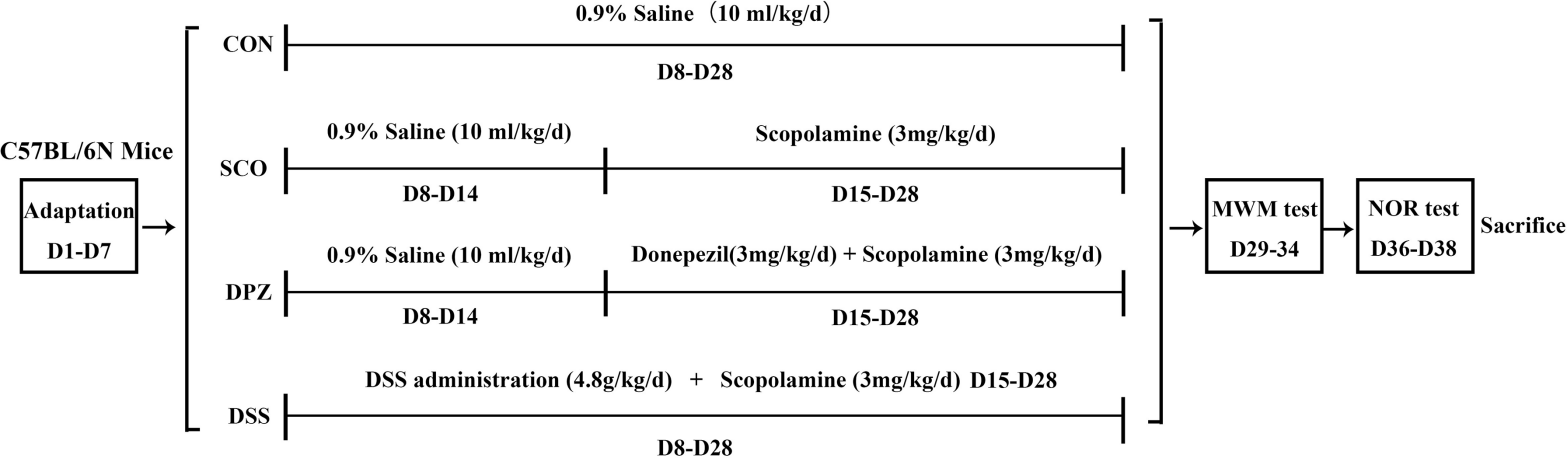
The diagram for the experimental design.

**Figure 2:**
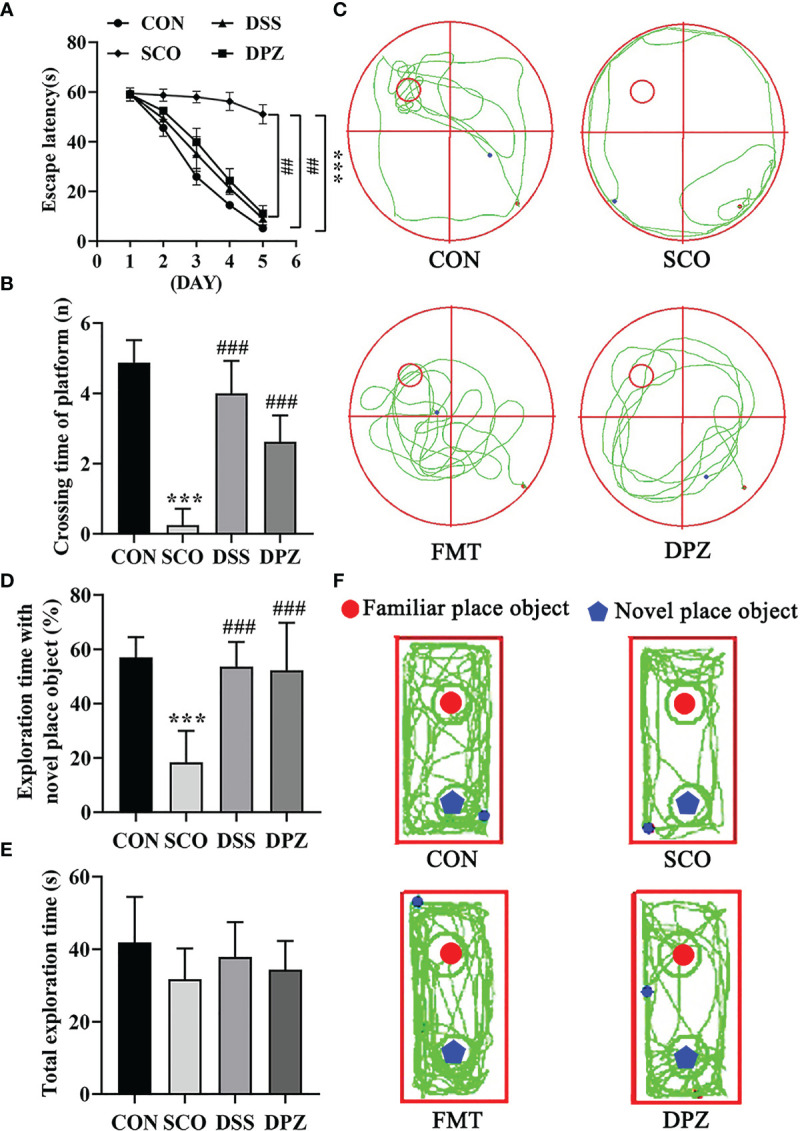
Evaluation of spatial learning and memory performance of CON, SCO, DSS, and DPZ 4 groups using the Morris water maze test and novel object recognition at week 2 after the commencement of their respective diets (n=8-10). For the **(A–C)** MWM test analysis, we see the following: **(A)** the escape latency time to reach the hidden platform during training days; **(B)** the number of entries in the platform zone during the probe trial; and **(C)** the representative track plots of 4 groups. For the **(D–F)** NOR analysis, we see the following: **(D)** the percentage of time spent with the object in the novel place to the total object exploration time; **(E)** the total object exploration time; and **(F)** the representative track plots of 4 groups: ^##^ *p* < 0.01, ***p < 0.001 vs. CON, ^###^ *p <* 0.001 vs. SCO.

To assess whether DSS treatment could prevent scopolamine-induced cognitive impairment in scopolamine-fed mice, we performed the Morris water maze test and novel object recognition (NOR) test (**Figure 1**), which explored hippocampus-dependent recognition memory and the ability to perform activities of daily living (**Figure 2**).

Mice were first tested for the acquisition and retention of spatial memory with the MWM test; the results are shown in **Figures 2A–C**. During the Morris water maze training, mice were set up to find a hidden platform beneath the surface of the water. In the acquisition phase, the escape latency to reach the platform gradually decreased during the training process in all groups (**Figure 2A**). Scopolamine-induced mice exhibited a significantly longer escape latency than did the CON group on days 5 (*p <* 0.001, **Figure 2A**).

Meanwhile, the test revealed that DSS dose treatment significantly decreased the escape latency compared to scopolamine-induced mice on days 5 (*p <* 0.01, **Figure 2A**). In the probe trial, the platform was removed, and the mice were placed into the quadrant opposite the target quadrant and allowed to swim freely for 60 s. Scopolamine-treated mice showed impaired memory, as evidenced by the significant decreases in the number of times crossing the target quadrant (**Figures 2B, C** **)**. However, DSS and DPZ treatment significantly decreased the number of times that scopolamine-induced mice crossed the target quadrant (*p <* 0.001, *p <* 0.001, respectively, **Figure 2B**).

NOR tests rely on rodents’ tendency to explore novel objects encountered within a dedicated environment that involves little stressful or aversive stimuli. As shown in **Figures 2D–F**, all groups exhibited similar total exploration time toward both objects. The scopolamine-induced mice exhibited a lower percentage of time spent with the novel object compared to total object exploration time (*p <* 0.001, **Figure 2D**), which indicated that restraint stress impaired the discriminative ability for the novel object. Compared with the scopolamine-induced mice, DSS and DPZ administration significantly improved the percentage of time spent with the novel object (*p <* 0.001, **Figure 2D**). However, total exploration time showed a non-significant difference in different groups (**Figure 2E**).

Taken together, these data suggest that DSS treatment reverses the impairment in spatial learning and memory induced by scopolamine.

### The Shift of Gut Microbiome in Mice Treated With DSS

**Figure 3:**
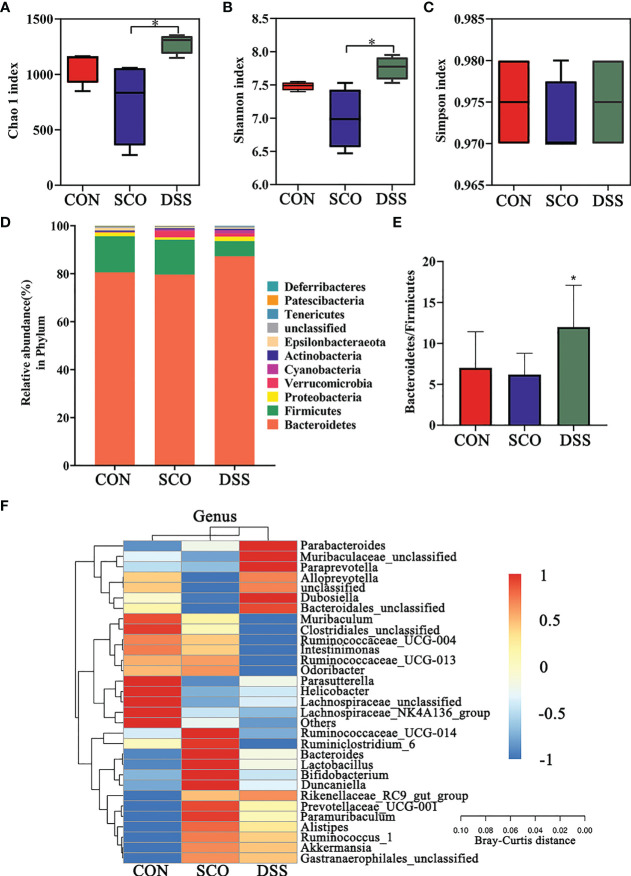
Evaluation of 16S rRNA gene sequence analyzes of the CON, SCO and DSS groups: **(A, B)** α-diversity Chao1 **(A)**, Shannon indices **(B)** and Simpson index **(C)** in each group, **(D)**. Correlation heatmap of the gut microbiota and metabolite phylum (n = 8). **(E)** The ratio of *Bacteroidetes/Firmicutes*. **(F)** Correlation heatmap of gut microbiota and metabolite genera (top 30 general analysis) (n = 8). *p < 0.05 vs. SCO.

To assess the effects of DSS administration on gut microbiota in mice, the fecal microbiota were analyzed using 16S rRNA gene sequence analyzes (**Figure 3**). However, donepezil has demonstrated some effects at the cellular and molecular system level associated with cognitive disorder in non-clinical studies. Therefore, donepezil was used as a positive control drug for the cognitive impairment induced by scopolamine in this study. In the behavioral test, the results already suggest that DSS-induced cognitive improvement is the same as the positive control. Next, the focus of this experiment is to analyze the effect of Danggui-Shaoyao-San treatment on the intestinal flora, so as to explore whether the compound can alleviate the cognitive impairment caused by scopolamine through the microbial–gut–brain axis. Thus, we chose not to use the donepezil group in the 16S rRNA gene sequence analysis.

In the evaluation of gut microbiome diversity, alpha diversity can reflect the abundance and diversity of species within a community. As shown in **Figures 3A–C**, the Chao1 and Shannon richness of the gut microbiota significantly increased in the DSS group compared with the SCO group, which means higher community stability (34) (*p <* 0.05, *p <* 0.05), and there were no significant differences in Simpson richness.

We calculated the relative abundances of each group at all kinds of classification levels, especially at the level of phylum and genus, to find the changes in the intestinal microbiota structure of different groups. Differences in relative abundances of the bacterial phylum and genus in the intestinal microbiota of subjects are shown in **Figures 3C, D**. The results identified that the dominant phyla were Bacteroidetes, Firmicutes, Proteobacteria, and Verrucomicrobia, together accounting for an average of 97.3%, 97.79% and 98.8% of all classifiable sequences in the CON, SCO and DSS groups, respectively. The DSS group had a higher abundance of Bacteroidetes, and the Bacteroidetes/Firmicutes ratio decreased compared to the SCO group (*p <* 0.05, **Figures 3D, E** **)**.

The results identified that the dominant genera were *Muribaculaceae* (S24-7), *Muribaculum*, *Bacteroides*, and *Alloprevotella*, together accounting for an average of 72.86%, 70.52%, and 76.94% of all classifiable sequences in the CON, SCO and DSS groups, respectively (**Figure 3F**). After DSS administration, the abundance of *Muribaculaceae*, *Alloprevotella*, *Parasutterella*, *Parabacteroides*, and *Akkermansia*, etc., increased which could improve lipid metabolic functionalities such as *Parasutterella*, which plays a role in cholesterol metabolism (35). In particular, *Muribaculaceae* are versatile with respect to complex carbohydrate degradation (36). *Akkermansia* can effectively repair the damaged integrity of the intestinal epithelium barrier and regulate dyslipidaemia in AD model mice (37). From the results, we demonstrated that DSS administration did modulate the abundance and diversity of gut microbiota which improved lipid metabolic functionalities.

### Effect of DSS on the Intestinal Pathology and Inflammation in Scopolamine-Induced Mice

**Figure 4:**
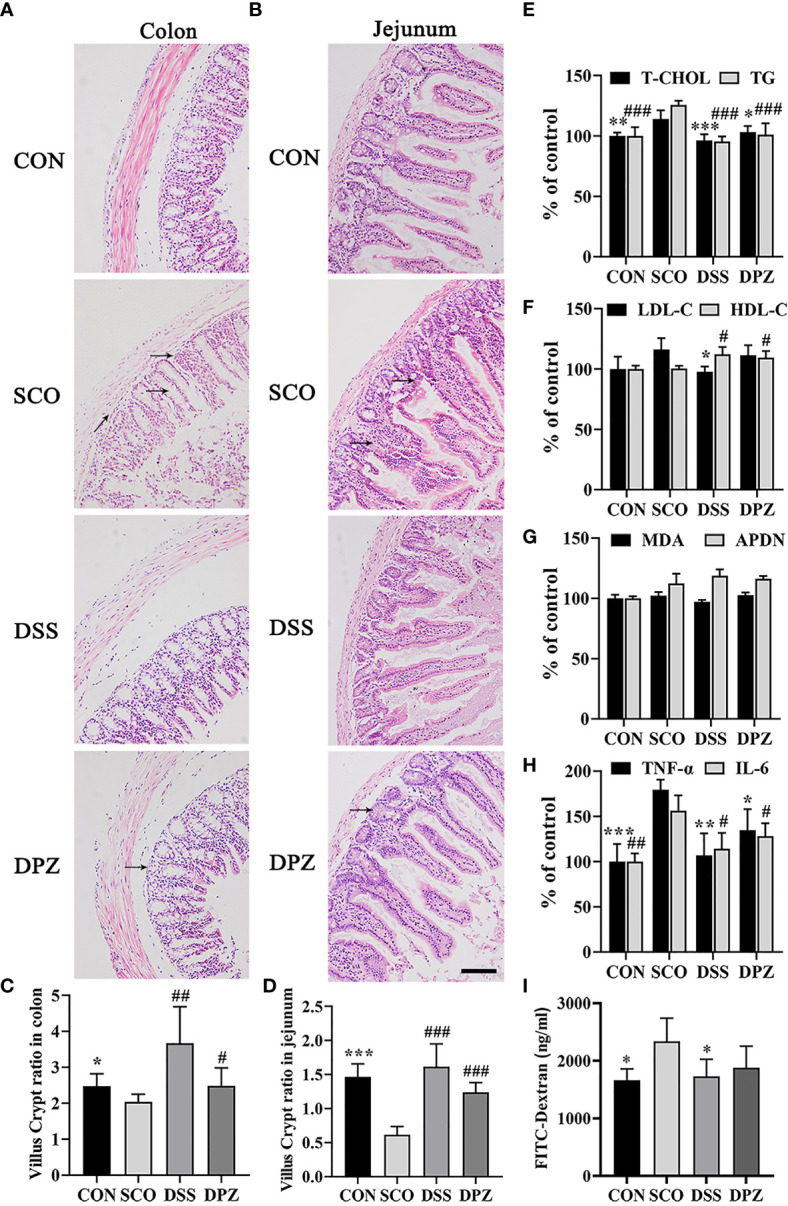
DSS ameliorated intestinal barrier impairment induced by scopolamine. H&E staining was performed to assess the morphology of the jejunum and colon collected from different groups and photographed under a microscope (magnification, ×10), bar = 200 μm. H&E staining images of **(A)** colon tissues and **(B)** jejunum tissues. The villus–crypt ratio in the jejunum **(C)** and colon **(D)**, *p < 0.05, **p < 0.01, ***p < 0.001 vs. CON, ^#^ *p* < 0.05, ^##^ *p* < 0.01, ^###^ *p* < 0.01 vs. SCO. **(E–I)** Inflammation cytokines and lipid levels in serum (n=3-5). **(E)** Total cholesterol (T-CHO) expression in the serum of the mice *p < 0.05, **p < 0.01, ***p < 0.001 vs. SCO; total glyceride (TG) expression in the serum of the mice ^###^ *p <* 0.001 vs. SCO, and expression in the serum of the mice. **(F)** Low-density lipoprotein cholesterol (LDL-C) expression in the serum of the mice *p 0.05 vs. SCO; high-density lipoprotein cholesterol (HDL-C) expression in the serum of the mice ^#^ *p <* 0.05 vs. SCO. **(G)** Adiponectin (APDN) and malondialdehyde (MDA) expression in the serum of the mice. **(H)** TNF-α expression in the serum of the mice. **(I)** FITC-dextran in the serum of the mice caused by scopolamine *p < 0.05, **p < 0.01, ***p < 0.001 vs. SCO; IL-6 expression in the serum of the mice ^#^ *p* < 0.05, ^##^ *p* < 0.01 vs. SCO.

To assess whether the DSS can suppress inflammatory responses in intestinal tissues of the mice and protect intestinal function, we proceeded with the histological evaluation of the colon and jejunal mucosa. The H&E staining of intestinal tissue directly reflects the injury of the intestinal epithelial. Indicated by the arrows in **Figures 4A, B**, the structure of the intestinal wall was damaged, which shows that the intestinal villus was fractured, shortened and atrophic along with the exposed lamina propria, as well as the loss of crypts and glands in the jejunum and colon of scopolamine-treated mice. However, DSS treatment resulted in significant attenuation of the jejunal and the colon lesions in the mice, such as less intestinal wall structure destruction (**Figures 4A, B** **)**. Moreover, the villus height/crypt depth ratios of the colon and jejunum were lower (p < 0.05, p < 0.001) in SCO groups and the villus crypt ratio of the colon and jejunum was both increased (p < 0.01, p < 0.001) in the DSS group compared to SCO group (**Figures 4C**, **D**). As shown above, the structure of the intestinal wall, which was damaged by the scopolamine, becomes clear, and damage of the intestinal epithelium and muscularis mucosae is repaired after DSS treatment.

In addition, DSS treatment could significantly decrease T-CHO and TG levels in the serum compared with the SCO group (*p <* 0.001, *p <* 0.001, **Figure 4E**). The results also indicated that DSS could increase HDL-C and decrease LDL-C expression in the serum of the scopolamine-treated mice (*p <* 0.05, *p <* 0.05, **Figure 4F**). Among all groups in the MDA and APDN levels (**Figure 4E**), there was no significant difference in serum.

Moreover, to investigate whether there were inflammation cytokines by scopolamine, intestinal TNF-a and IL-6 were examined. Scopolamine treatment significantly increased the TNF-a and IL-6 expression in the intestines (**Figure 4G**). The administration of DPZ decreased the levels of TNF-a and IL-6 in the intestines compared with the SCO group mice. A more obvious downregulation in TNF-a and IL-6 levels was also found in the DSS group mice. These results indicate that the overexpression of TNF-a and IL-6 may impair intestinal barrier function, while DSS treatment may inhibit necrotic and ulcerative lesions (*p <* 0.05, *p <* 0.001, **Figure 4H**). Next, to evaluate the severity of intestinal barrier dysfunction, small fluorescent probes (fluorescein isothiocyanate FITC-dextran) and the spectrophotometric quantification of the plasma dextran level were measured as indicators of intestinal permeability. As shown in **Figure 4G**, compared with the CON group, the dextran in the serum was both significantly increased in the scopolamine-treated group. The DSS and DPZ groups had significantly lower levels of the serum dextran than the SCO groups. These data suggest that it was able to increase intestinal permeability of FITC-dextran in the mice caused by scopolamine (**Figure 4I**). Notably, after the treatment of DSS, the plasma dextran level clearly decreased.

### DSS Restored Dementia Related Morphological Anomalies in the Hippocampus

**Figure 5:**
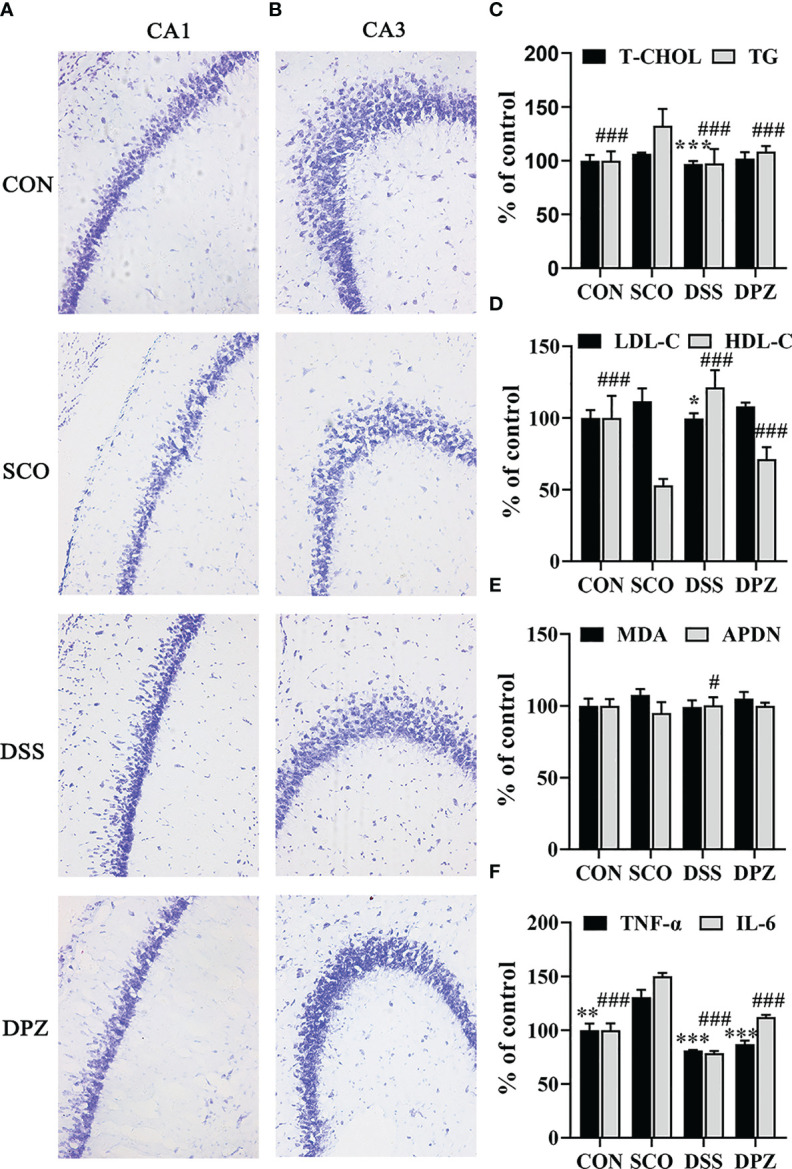
DSS restored dementia-related morphological anomalies in the hippocampus. Evaluation of the number of surviving neuronal cells in mice by Nissl’s staining. **(A)** Representative microphotographs of the Nissl’s stained CA1 area of hippocampal sections from the 4 experimental groups at ×20 magnification. Nissl bodies were dyed deep blue, and the cell nuclei were lightly stained. **(B)** Representative microphotographs of the Nissl-stained CA3 area of hippocampal sections from the 4 experimental groups at ×20 magnification, bar = 100 μm. The image represents 1 mouse. The studies were repeated twice in another 2 mice. **(C–F)** Inflammation cytokines and lipid levels in the brain (n=3-5). **(C)** Total cholesterol (T-CHO) expression in the brain of the mice ***p < 0.001 vs. SCO; total glyceride (TG) expression in the brain of the mice ^###^ *p <* 0.001 vs. SCO. **(D)** Low-density lipoprotein cholesterol (LDL-C) expression in the brain of the mice *p < 0.05 vs. SCO; high-density lipoprotein cholesterol (HDL-C) expression in the brain of the mice ^###^ *p <* 0.001 vs. SCO. **(E)** Malondialdehyde (MDA) and adiponectin (APDN) ^#^ *p <* 0.05 vs. SCO expression in the brain of the mice. **(F)** TNF-α expression in the brain of the mice **p < 0.01, ***p < 0.001 vs. SCO; IL-6 expression in the brain of the mice ^###^ *p <* 0.001 vs. SCO.

In order to evaluate the effect of DSS treatment in the hippocampal CA1 and CA3 regions, a representative Nissl’s staining was performed to examine the hippocampus’ histological changes in the 4 groups (**Figures 5A, B** **)**. According to Nissl staining, arrows indicated that the neurons of the hippocampus in CON group mice were in a large quantity and in a compact arrangement, while neurons in the SCO group were sparsely arranged and the morphology of the Nissl-stained cell bodies was abnormal. However, no remarkable neuronal loss in CA1 and CA3 region of the hippocampus was observed in mice after DSS treatment, as compared with the CON group. On the contrary, DSS treatment improved the abnormal morphology of Nissl-stained cell bodies in the CA1 region and in the CA3 region compared with the SCO group. Above results indicated that DSS restored the anomalies in morphology caused by scopolamine.

Meanwhile, the LDL-C level in brain tissues decreased (*p <* 0.05, **Figure 5D**) after DSS feeding, whereas the HDL-C levels markedly increased (*p <* 0.05, **Figure 5E**). Interestingly, DSS treatment could significantly decrease the T-CHO and TG levels in the brain tissues, compared with those in SCO group (*p <* 0.001, *p <* 0.001, respectively, **Figure 5C**). Furthermore, the results also indicated that DSS could increase ADPN in the brain tissues in the scopolamine-induced mice (*p <* 0.05, **Figure 5E**). Among all groups in the MDA level, there was no significant difference in serum and brain tissues. There were no significant differences in the MDA of brain levels.

Nest, to investigate whether there were inflammation cytokines by scopolamine, hippocampal TNF-a and IL-6 were examined. Scopolamine treatment significantly increased the TNF-a and IL-6 expression in the hippocampus (*p <* 0.001, **Figure 5F**). Administration of DPZ decreased the levels of TNF-a and IL-6 in the hippocampus compared with the SCO group mice. A more obvious downregulation in TNF-a and IL-6 levels was also found in the DSS group mice. These results indicate that the overexpression of TNF-a and IL-6 may impair hippocampal-dependent memory, while DSS treatment may inhibit pathological changes of hippocampal morphology.

### Scopolamine-Induced Amnesia Changes Are Reversed by FMT Treatment

**Figure 6:**
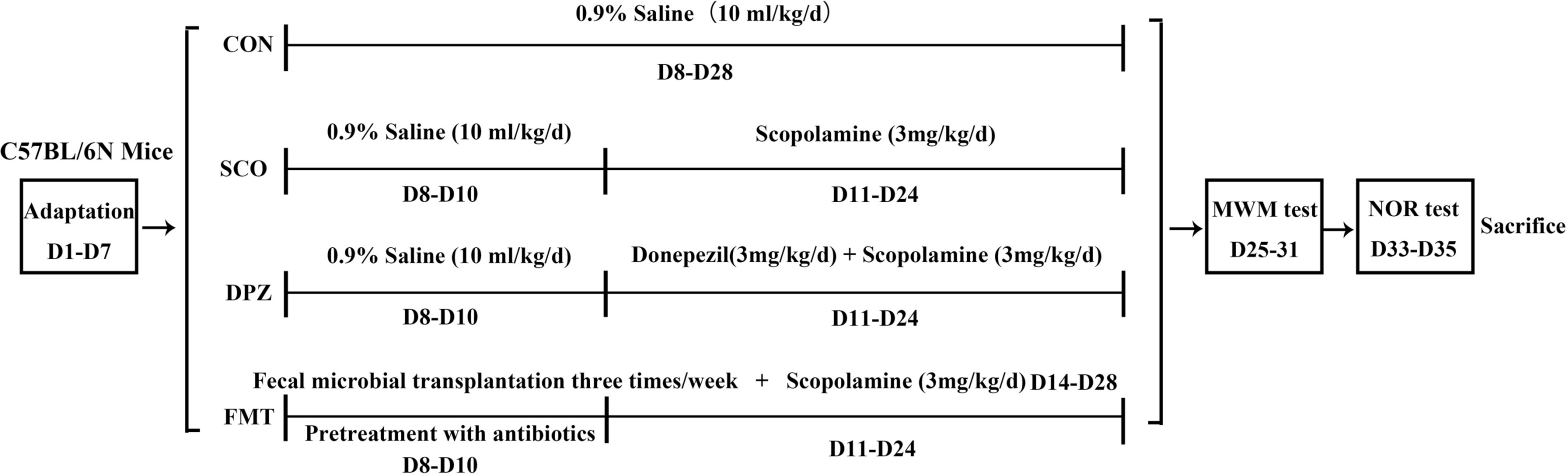
The diagram for the experimental of FMT design.

**Figure 7:**
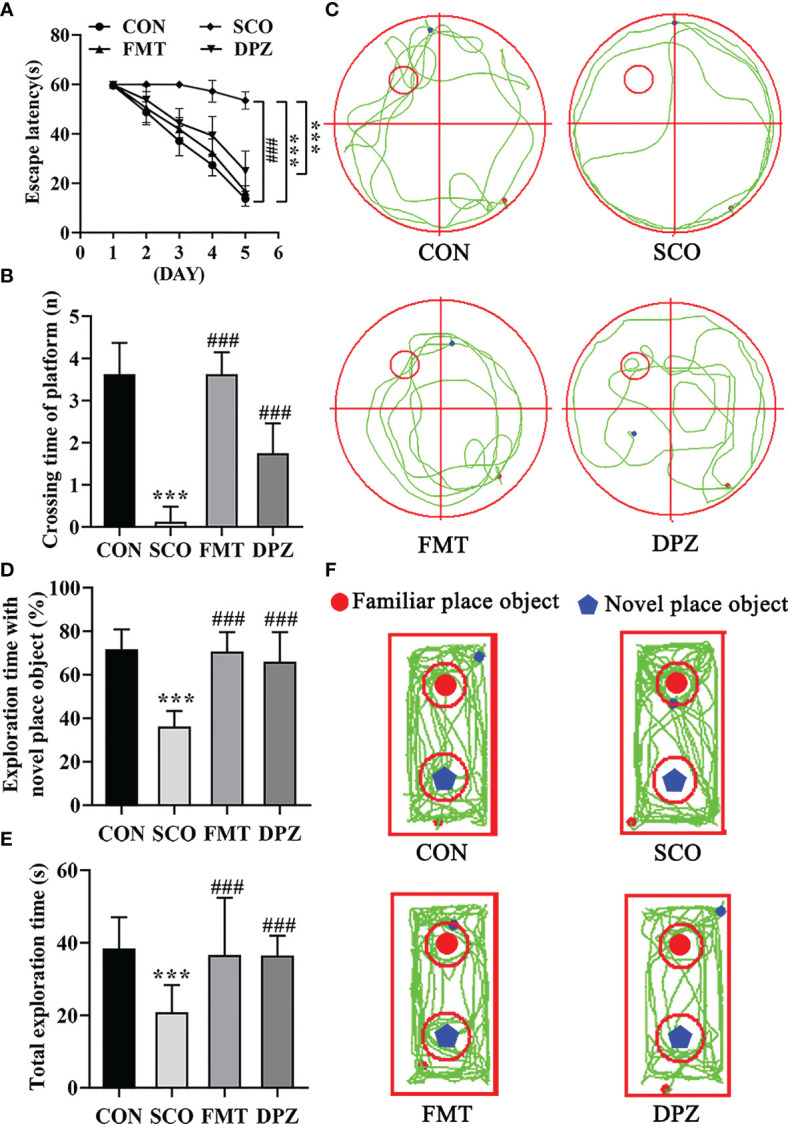
Evaluation of spatial learning and memory performance of the CON, SCO, FMT and DPZ groups using the Morris water maze test and novel object recognition (n=8-10). For **(A–C)** MWM test analysis, we see the following: **(A)** the escape latency time to reach the hidden platform during training days; **(B)** the number of entries in the platform zone during the probe trial; and **(C)** the representative track plots of 4 groups. For D-F NOR analysis, we see the following: **(D)** the percentage of time spent with the object in the novel place to total object exploration time; **(E)** the total object exploration time; and **(F)** the representative track plots of the 4 groups: **p < 0.01, ***p < 0.001 vs. CON, ^##^ *p* < 0.01, ^###^ *p* < 0.001 vs. SCO.

We performed two behavioral tests and designed four groups, CON, SCO, DPZ and FMT groups (**Figure 6**), to explore whether DSS treatment could attenuate cognitive impairment *via* the microbiota–gut–brain axis (**Figure 7**).

Mice were first tested for the acquisition and retention of spatial memory with the MWM test; the results are shown in **Figures 7A–C**. In the acquisition phase, the escape latency to reach the platform gradually decreased during the training process in all groups (**Figure 7A**). FMT treatment exhibited significantly the shorter escape latency compared to scopolamine-induced mice on day 5 (*p <* 0.001, **Figure 7A**). In the probe trial, scopolamine-treated mice showed impaired memory, as evidenced by the significant decreases in the number of times crossing the target quadrant (*p <* 0.001, **Figures 7B, C** **)**. However, FMT treatment significantly decreased the number of times that scopolamine-induced mice crossed the target quadrant (*p <* 0.001, **Figure 7B**). In NOR tests, FMT administration significantly improved the percentage of time spent with the novel object compared with the scopolamine-induced mice (*p <* 0.001, **Figures 7D, F**) and FMT groups exhibited a longer total exploration time toward both objects (*p <* 0.001, **Figure 7E**). Taken together, these data suggest that FMT treatment reverses the impairment in spatial learning and memory induced by scopolamine.

### The Shift of Gut Microbiome in Mice Treated With FMT

**Figure 8:**
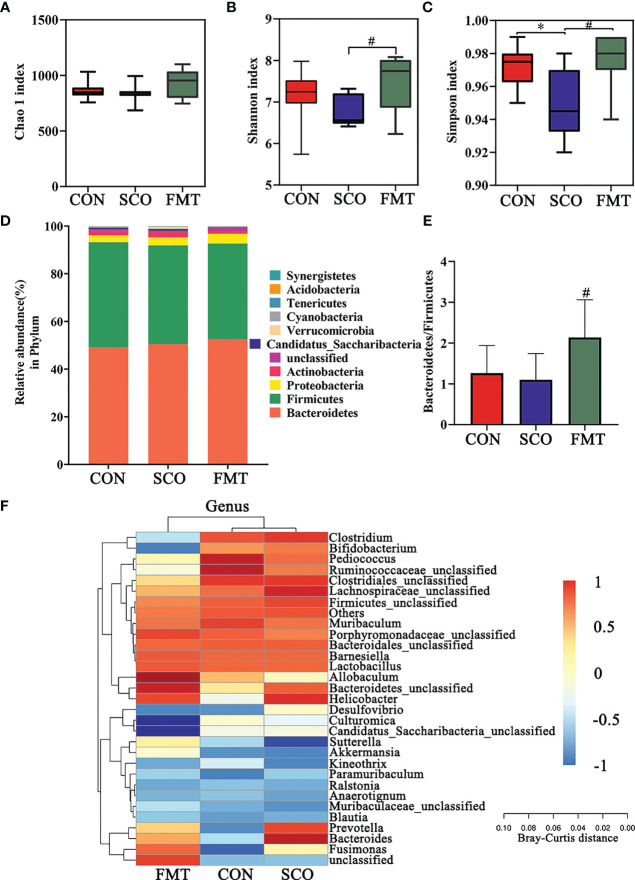
Evaluation of 16S rRNA gene sequence analyzes of the CON, SCO and FMT groups: **(A, B)** α-diversity Chao1 **(A)**, Shannon and Simpson index **(C)** in each group; **(C)** clustering analysis of different metabolites in each group dominant phylum; and **(D)** correlation heatmap of gut microbiota and metabolite phylum (n = 8). **(E)** The ratio of *Bacteroidetes*/*Firmicutes*. **(F)** Correlation heatmap of gut microbiota and metabolite genera (top 30 general analysis) (n = 8). *p < 0.05 vs. CON, ^#^ *p <* 0.05 vs. SCO.

To assess the effects of FMT administration on gut microbiota in mice, fecal microbiota was analyzed using 16S rRNA gene sequence analyzes (**Figure 8**).

In the evaluation of gut microbiome diversity, alpha diversity can reflect the abundance and diversity of species within a community. As shown in **Figures 8A–C**, the Shannon richness and Simpson richness of the gut microbiota significantly increased in the FMT group compared with the SCO group, which means a higher community stability (34) (*p <* 0.05, *p <* 0.05), and there were no significant differences in Chao 1 richness.

We calculated the relative abundances of each group at all kinds of classification levels, especially at the level of phylum and genus, to find the changes in intestinal microbiota structure of different groups. Differences in relative abundances of bacterial phylum and genus in the intestinal microbiota of subjects are shown in **Figures 8C, D**. The results identified that dominant phyla were Bacteroidetes, Firmicutes, Proteobacteria and Actinobacteria, together accounting for an average of 98.01%, 97.37% and 97.63% of all classifiable sequences in the CON, SCO and FMT groups, respectively (**Figure 8F**). The FMT group had a higher abundance of Bacteroidetes, and the Bacteroidetes/Firmicutes ratio increased compared to the SCO group (*p <* 0.05, **Figures 8D, E** **)**.

The results identified that the dominant genera were *Bacteroidales*_unclassified, *Barnesiella*, *Allobaculum*, *Pediococcus*, *Lactobacillus*, *Muribaculum* and *Clostridiales_unclassified* together accounting for an average of 64.72%, 57.32% and 62.95% of all classifiable sequences in the CON, SCO and FMT groups, respectively. After FMT administration, the abundance of *Allobaculum*, *Muribaculaceae*, *Bacteroidetes*, *Parabacteroides* and *Akkermansia*, etc., increased which could improve lipid metabolic functionalities such as *Allobaculum*. Furthermore, the relative abundance of *Allobaculum* in mice and rats has been correlated with aging, high-fat diets and fatty acid metabolism, and it has been reported that *Allobaculum* is an active glucose utiliser and producer of lactate and butyrate (38). In particular, the same results as those of DSS group *Muribaculaceae* and *Akkermansia* improved in FMT groups which can effectively repair the damaged integrity of the intestinal epithelium barrier and regulate dyslipidaemia in cognitive disorder mice (37). From the results, we demonstrated that FMT administration did modulate the abundance and diversity of gut microbiota which improved lipid metabolic functionalities.

### DSS Treatment Ameliorated the Barrier Function of Intestine the Degradation of TJ Proteins Induced by Scopolamine

**Figure 9:**
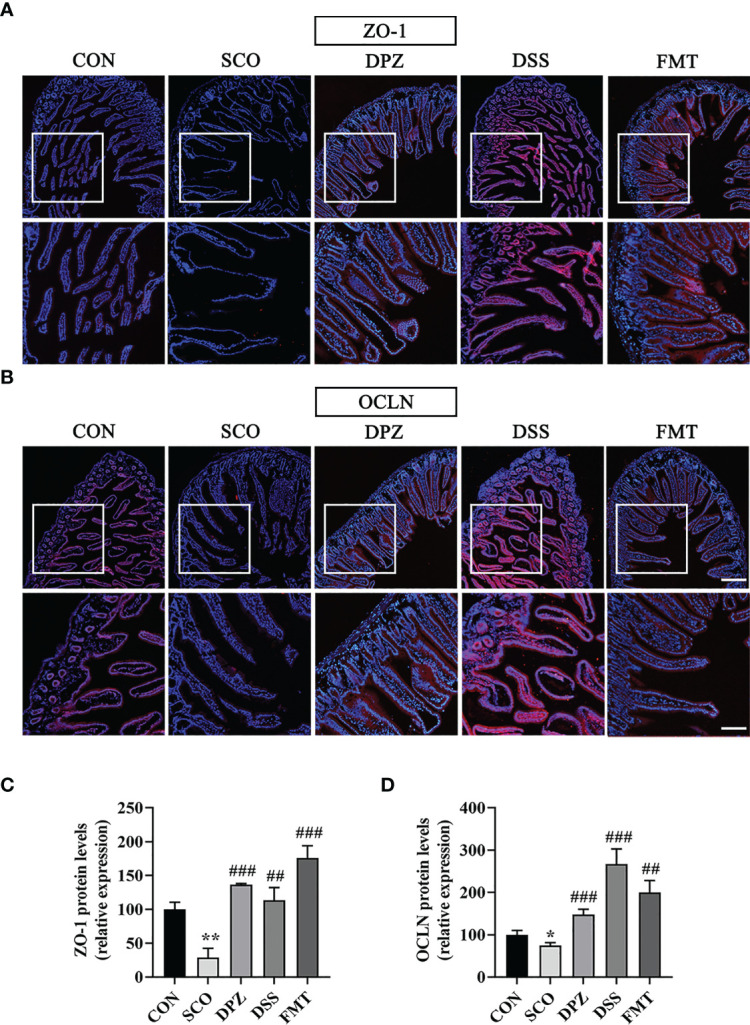
The protective effects of **(A, B)** on the intestinal barrier. For immunofluorescence staining of ZO-1, OCLN (red colour) in jejunum tissue, stained slides with the CON, SCO, DPZ, DSS and FMT groups. Immunofluorescence staining of ZO-1 **(A)** and OCLN **(B)** was observed under a fluorescence microscope at ×10 magnification (bar = 200μm) and ×20 magnification (bar = 100μm). ZO-1 **(C)** and OCLN **(D)** was represented by fluorescence quantitative statstics (n =3), *p < 0.05, **p < 0.01, vs. CON, ^##^ *p* < 0.01, ^###^ *p* < 0.001, vs. SCO.

**Figure 10:**
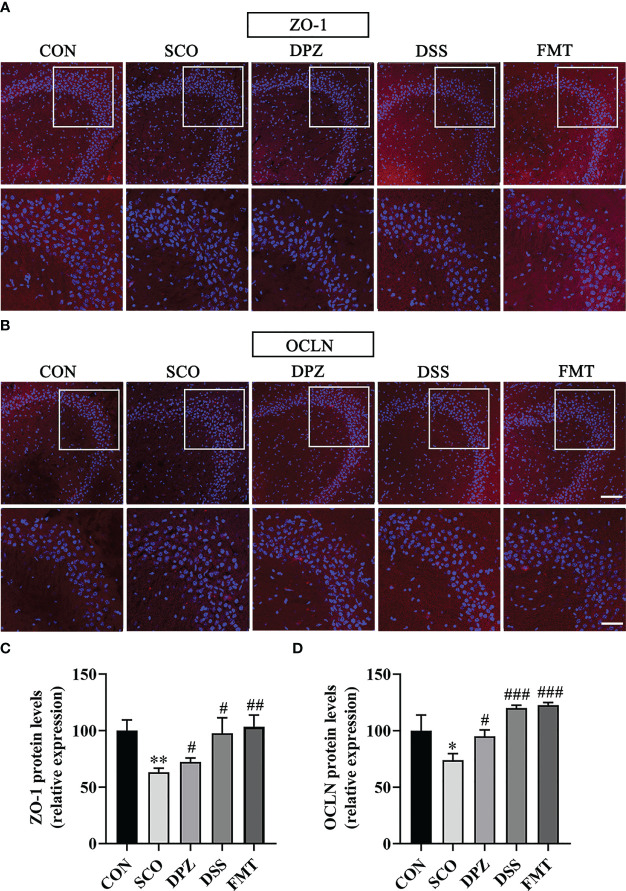
The protective effects of **(A, B)** on the hippocampus. Immunofluorescence staining of ZO-1 **(A)** and OCLN **(B)** was observed under a fluorescence microscope at ×20 magnification (bar = 100μm) and ×40 magnification (bar = 50μm). ZO-1 **(C)** and OCLN **(D)** were represented by fluorescence quantitative statistics. (n = 3) *p < 0.05, **p < 0.01, vs. CON, ^#^ *p* < 0.05, ^##^ *p* < 0.01, ^###^ *p* < 0.001 vs. SCO.

Tight junction (TJ) proteins are a cell-adhesion compound that acts as a crucial barrier function of epithelia and endothelia. The protein intensitiesy of OCLN and ZO-1 in the gut were investigated through immunofluorescence and Western blot in order to further study the role of DSS in repairing intestinal barrier function. On the one hand, double immunofluorescence staining results showed that the morphology and expression of TJ proteins were disrupted and lost in the scopolamine-induced group (ZO-1: *p* < 0.01; OCLN: *p* < 0.05, *p* < 0.001), while the DSS administration group prevented the degradation of TJ proteins and maintained intestinal barrier integrity (ZO-1: *p* < 0.01; OCLN: *p* < 0.001 **Figures 9A–D**). Likewise, the FMT group also greatly protected the intestinal barrier by intensifying the protein concentration of OCLN and ZO-1 (ZO-1: *p* < 0.001; OCLN: *p* < 0.01 **Figures 9A–D**). On the other hand, the WB results showed that the DSS as well as the FMT groups could enhance the expression of ZO-1 and OCLN compared to the SCO group (ZO-1: *p* < 0.05; OCLN: *p* < 0.01, *p* < 0.05 **Figure 10C**).

### DSS Treatment Ameliorated Scopolamine-Induced Blood–Brain Barrier Disruption

**Figure 11:**
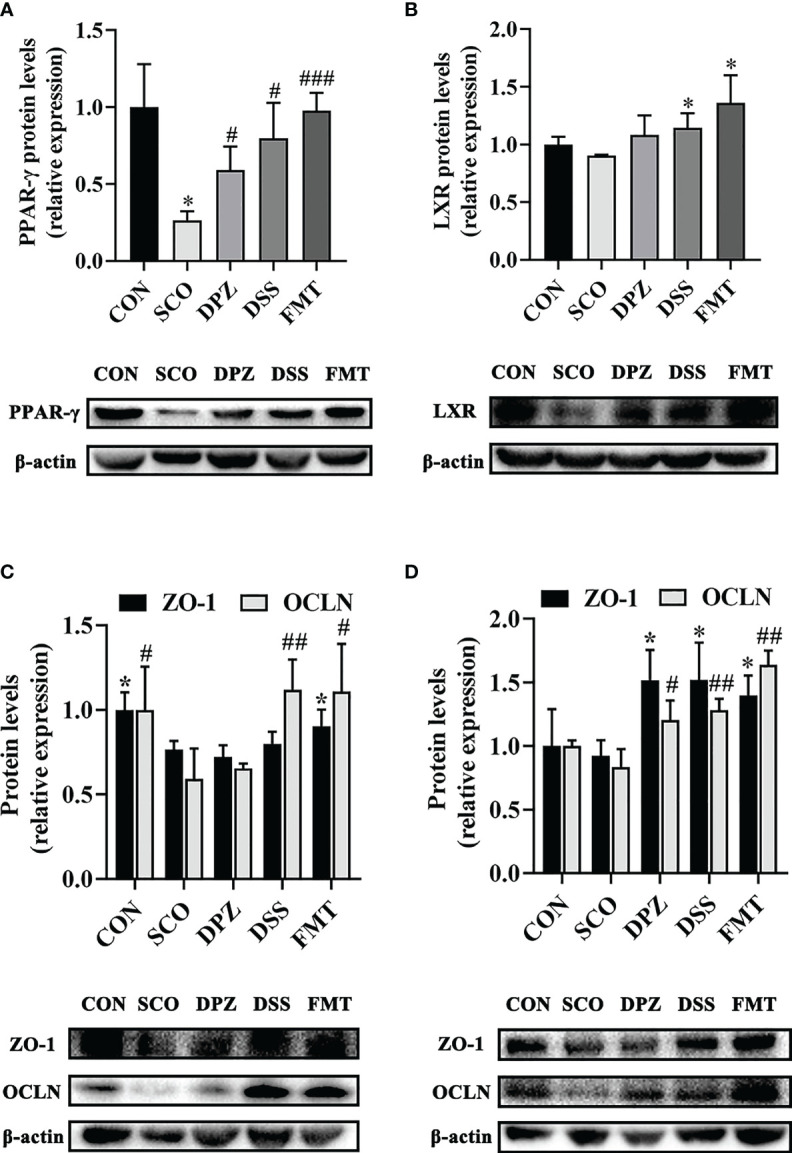
Western blot analysis shows the expression of PPAR-γ **(A)**, ZO-1 and OCLN **(C)** in gut and LXR **(B)** together with ZO-1 and OCLN **(D)** in hippocampus tissues of each experimental group. Densitometry analysis of the Western blot band of PPAR-γ, LXR, ZO-1 and OCLN used Quantity One (n = 3). The relative ratio of PPAR-γ, LXR, ZO-1 and OCLN was represented by densitometric analysis. *p < 0.05, vs. CON; ^#^ *p <* 0.05, ^###^ *p <* 0.001 vs. SCO in PPAR-γ R; *p < 0.05 vs. SCO in LXR; *p < 0.05 vs. SCO in ZO-1, ^#^ *p <* 0.05, ^##^ *p <* 0.01 vs. SCO in OCLN.

The blood–brain barriers are the crucial lines of defence against harmful external stimulus. These host barriers consist of epithelial and endothelial cells which are connected to each other by tight-junction proteins along with several other supporting structures. To further study the role of DSS in maintaining BBB integrity, changes in TJ (ZO-1 and OCLN) proteins in the brain were detected by immunofluorescence staining and WB. Immunofluorescence staining results were as shown in **Figure 10**; the SCO group significantly downregulated the expression of ZO-1 and OCLN compared with the CON group (*p <* 0.01, *p <* 0.05, **Figures 10A–D**). DPZ, DSS and FMT treatments could significantly upregulate the expression of ZO-1 and OCLN compared with the scopolamine group (ZO-1: *p <* 0.05, *p <* 0.05, *p <* 0.01; OCLN: *p <* 0.05, *p <* 0.001, *p <* 0.001, **Figures 10C, D** **)**. Simultaneously, the WB results showed that compared with the scopolamine group, the DPZ, DSS and FMT groups could significantly upregulate the expression of ZO-1 and OCLN (ZO-1: *p <* 0.05, *p <* 0.05, *p <* 0.05; OCLN: *p <* 0.05, *p <* 0.01, *p <* 0.01, **Figure 11C, D**). However, there were no differences in the SCO group compared to the CON group.

### DSS Regulated Lipid Metabolism *via* the PPAR-γ/LXR Pathway in Gut–Brains After Scopolamine Injection

LXR is one of the major players of lipid metabolism, mainly recognised for its role as a cholesterol sensor and promoting the loss of cellular cholesterol, while peroxisome proliferator activated receptor gamma (PPAR-γ) signalling is a major element in lipid metabolism. Research has shown that PPAR-γ is present at high levels in adipose tissue, brain and in particular the gut. Moreover, PPAR-γ is a butyrate sensor in the colonic lumen (39). In our study, we subsequently evaluated PPAR-γ expression in the colon as well as the LXR expression in the brain. The results show that DSS treatment displayed a significant increase, not only in PPAR-γ protein expression (*p <* 0.05, **Figure 11A**) but also in LXR (*p <* 0.01, **Figure 11B**). PPAR is interconnected with the functions of LXR in lipid metabolism (40). In addition, there are also studies to prove that agonists of LXR and PPAR-γ act to ameliorate dementia-related cognitive impairment and amyloid accumulation in murine models of AD (41). Compared with the control mice, PPAR-γ protein expression levels increased in the DPZ, DSS and the FMT group (p < 0.05, p < 0.05, p < 0.001), which showed a tendency to decreased values after scopolamine administration. Additionally, the protein expression of LXR was significantly increased in the DSS and FMT groups compared to the SCO group (*p <* 0.05, p < 0.05), but there were no differences in the SCO group compared to the CON group. Therefore, DSS treatment demonstrated significantly improved cognitive impairment, which may regulate lipid metabolism by activating LXR and PPAR-γ and alleviate cognitive disorder.

## Discussion

In this present study, we demonstrated a range of beneficial effects of DSS on cognitive impairment behaviors of mice in the SCO group *via* the gut–microbiota–brain axis and evaluated the mechanisms. For the first time, we provided new experimental evidence that DSS attenuated cognitive impairment *via* the microbiota–gut–brain axis assessed behaviorally and at the level of the hippocampus and prevented with the regulation of lipid metabolism and mucosal barrier dysfunction assessed with a broad range of techniques. Furthermore, by the use of FMT intervention, the FMT-induced improvement in cognitive function highlights the crucial role of the gut–microbiota–brain axis to mediate cognitive function and behavior in DSS.

Then, we chose a scopolamine-induced amnesia model in mice which is widely used to study neurological disorders that negatively impact learning and memory such as AD (42). It’s observed by modelling NOR and MWM tests that DSS treatment reverses the impairment in spatial learning and memory in scopolamine-induced mice. After administering scopolamine for 2 weeks, the Morris water maze test showed scopolamine-induced amnesic behavior. On the contrary, DSS significantly reduced amnesic behavior in both tests. We also found that DSS treatment significantly decreased the number of times that scopolamine-induced mice crossed the target quadrant at MWM test analysis. Moreover, DSS administration significantly improved the percentage of time spent with the novel object. Furthermore, we performed a representative Nissl staining to examine the hippocampus’ histological changes, indicating that DSS restored amnesia-related morphological anomalies in the hippocampus.

A growing body of evidence suggests the hypothesis that the microbiota–gut system can be thought of as a single unit that interacts with the brain *via* the microbiota–gut–brain axis. Through this axis, a constant interplay mediated by several products originating from the microbiota guarantees the physiological development and shaping of the gut and the brain (43, 44). We evaluated gut microbiome diversity and calculated the relative abundances of each group at all kinds of classification level, especially at the level of phylum and genus, to find the changes in the intestinal microbiota structure of different groups. By using 16S rRNA gene sequencing analysis, we found that DSS consumption ameliorated a shift of gut microbiota composition induced by scopolamine. At same time, the diversity and richness of the gut microbiota were significantly increased, and the Bacteroidetes to Firmicutes ratio dramatically increased. Firmicutes generated more harvestable energy than Bacteroidetes. Relatively high ratios of Bacteroidetes to Firmicutes not only influence carbohydrate metabolism, but also alter the production of short-chain fatty acids (45). Furthermore, the results identified that the abundance of Muribaculaceae, Alloprevotella, Parasutterella, Parabacteroides, Akkermansia, etc., increased with DSS administration which could improve lipid metabolic functionalities such as Parasutterella, which plays a role in cholesterol metabolism (35). In particular, Muribaculaceae are versatile with respect to complex carbohydrate degradation (36). Akkermansia can effectively repair the damaged integrity of the intestinal epithelium barrier and regulate dyslipidaemia in dementia model mice (37). Overall, these findings support that showed that DSS administration did change the specific composition and function of gut microbiota and modulate the abundance and diversity of gut microbiota and served as an important regulator for lipid metabolic and intestinal epithelium barrier.

Given this background, we further tested the level of lipids and inflammation in serum and brain tissues. We found that DSS treatment mice showed a significantly decreased inflammatory factor, along with reducing lipid metabolism disorders, such as decreased high levels of total cholesterol, triglycerides, and low-density lipoprotein, while increased high-density lipoprotein compared with scopolamine-induced mice. It is important to note that DSS administration increases the ADPN in the brain which is known to regulate various metabolic functions and reduces inflammation, treating blood–brain barrier breakdowns in AD (46). Furthermore, we detected the fluorescence intensity together with protein expression of tight-junction markers ZO-1 and OCLN, through histological analysis of the intestine and FITC-dextran detection, finding DSS supplementation amelioration at the level of the intestine not only in intestinal permeability and also in the mucosal barrier.

A critical objective was to determine whether DSS treatment could attenuate cognitive impairment *via* the microbiota–gut–brain axis in scopolamine-induced amnesia, and fecal microbial transplantation (FMT) was performed in C57BL/6N mice to support our viewpoint. The results of behavioral tests and 16S rRNA gene sequence analyzes supported the FMT-induced improvement in cognitive function which highlights the role of the gut–microbiota–brain axis to mediate cognitive function and behavior, thereby suggesting that the microbiota–gut–brain axis is considered to be a key regulator of neural function.

To further verify how microbiota alter the gut–brain axis, the protein content of ZO-1 and OCLN in the brain was detected. The blood–brain barrier impairment is recognised as a critical factor contributing to Alzheimer’s disease pathogenesis (47). To maintain a sealed environment for the brain, the BBB relies on tight junctions which comprise a number of proteins. The tight junctions (TJs) are key players in the control of blood–brain barrier (BBB) properties; among them, ZO-1 and OCLN have been shown to be the key transmembrane proteins that regulate endothelial barrier integrity. The immunofluorescence and WB results showed that the ratio of ZO-1 and OCLN was significantly reduced in the SCO group compared with the CON group, and this effect was significantly reversed in the DSS and FMT administration group, indicating that DSS and FMT can decrease the brain endothelial permeability and reinforce the blood–brain barrier. The gut barrier and blood–brain barrier represent a crucial line of defence to protect underlying structures against harmful external stimuli. Therefore, it can be seen from this that DSS improves intestinal barrier and blood–brain barrier function *via* the microbiota–gut–brain axis.

Another critical objective was to determine how DSS attenuates cognitive impairment *via* the microbiota–gut–brain axis with regulation of lipid metabolism.

Peroxisome proliferator-activated receptor gamma (PPAR-γ) is present at high levels in adipose tissue, the brain and particularly in the intestine. PPAR-γ activated signalling has been reported to prevent dysbiotic expansion of pathogenic bacteria by driving the energy metabolism of colonic epithelial cells (48). Stimulating PPAR-γ can repair intestinal epithelial barrier damage by activating the inflammasome, contributing to the inhibition of neuroinflammatory response and neuronal loss (49). Furthermore, PPAR-γ agonist pioglitazone protected against scopolamine-induced cholinergic system deficit, including reduced acetylcholine levels, decreased choline acetyltransferase activity and increased acetylcholinesterase activity in the hippocampus or cortex (50). And correspondingly, in our study, we found that repeated scopolamine-induced dementia caused a reduction in PPAR-γ expression and treatment with the DSS and the FMT produced significant changes in the colon, compared with the scopolamine-induced group. Moreover, most of these receptors respond to lipid metabolites such as eicosanoids (PPARs) and liver X receptors (LXRs).

Next, our findings are supported by a growing body of evidence which shows that DSS treatment could significantly decrease the T-CHO, LDL-C and TG levels in the serum compared with the scopolamine-treated mice which displayed a significant increase in PPAR-γ protein expression. It could be proposed that DSS treatment affects cognitive disorder by activating PPAR-γ, thereby repairing the intestinal barrier and improving dyslipidaemia.

It has previously been demonstrated that activation of liver x receptor (LXR) improved cognition in Alzheimer’s disease (AD) mice by alleviating AD pathology (51). In addition, LXR has been shown to serve as a primary sensor of lipid metabolic cues, regulating lipid metabolism by repressing the transcriptional activation of enzymes involved in *de novo* lipogenesis and bile acid homeostasis. As a consequence of stimulating by LXR, lipid metabolism in the brain becomes improved. Besides, we observed increased LXR levels in scopolamine-induced mice which were restored with DSS treatment. Additionally, DSS treatment could significantly decrease the T-CHO and TG levels in the brain tissues, thus improving lipid metabolism disorder.

Apart from that, our study reveals an increase in LXR in the brain of DPZ, DSS and FMT-treated mice. Since LXR are transcription factors that control the expression of gene products involved in cholesterol homeostasis and several direct LXR target genes are intimately linked to cholesterol transport. Thus, we propose a guess that the link between PPAR-γ and lipid metabolism likely involves the nuclear receptor, such as LXR. Another study has shown that sustained PPAR or LXR activation results in amelioration of AD-related pathophysiology in Alzheimer’s disease model mice (52).

Moreover, a previous study demonstrated that the regulation of the PPAR-γ-LXR-APOE cascade may represent a significant molecular connection between adipocyte TG and cholesterol homeostasis. Therefore, it may regulate lipid metabolism by activating LXR and PPAR-γ and alleviate cognitive impairment pathology, thus improving cognitive dysfunction. These findings suggest that the ameliorative effect of DSS treatment on cognitive function can be attributed to the repair of metabolic disorders and the intestinal and blood–brain barriers, which might be by activating the LXR and PPAR-γ.

According to our research, we provided new experimental evidence that DSS may attenuate cognitive impairment *via* the microbiota–gut–brain axis with regulation of lipid metabolism in scopolamine-induced amnesia underlying this effect which would facilitate the development of new therapeutics for dementia.

## Data Availability Statement

The datasets presented in this study can be found in online repositories. The name of the repository and accession number can be found as follows: National Center for Biotechnology Information (NCBI) BioProject, https://www.ncbi.nlm.nih.gov/bioproject/, PRJNA819868.

## Ethics Statement

The animal study was reviewed and approved by the Animal Experiment Ethics Committee of Guangzhou University of Chinese Medicine. Written informed consent was obtained from the owners for the participation of their animals in this study.

## Author Contributions

Conducted experimental research, data analysis, draft writing and revision of the paper, PL and XZ. Performed experimental investigation, HZ. Data Curation, XW and RW. Project guidance and financial support, WZ, DY and QW. Project design, WL. All authors contributed to the article and approved the submitted version.

## Funding

This work was supported by the National Natural Science Foundation of China (No. 81973919) and No. (82074505), the National Natural Science Foundation of Guangdong province (No. 2019A1515011299) and Guangdong province science and technology plan international cooperation project (No. 2020A0505100052).

## Conflict of Interest

The authors declare that the research was conducted in the absence of any commercial or financial relationships that could be construed as a potential conflict of interest.

## Publisher’s Note

All claims expressed in this article are solely those of the authors and do not necessarily represent those of their affiliated organizations, or those of the publisher, the editors and the reviewers. Any product that may be evaluated in this article, or claim that may be made by its manufacturer, is not guaranteed or endorsed by the publisher.
